# Photodynamic and photobiological effects of light-emitting diode (LED) therapy in dermatological disease: an update

**DOI:** 10.1007/s10103-018-2584-8

**Published:** 2018-07-14

**Authors:** Elisabetta Sorbellini, Mariangela Rucco, Fabio Rinaldi

**Affiliations:** 1 International Hair Research Foundation (IHRF), Milan, Italy; 2 Human Advanced Microbiome Project-HMAP, Milan, Italy

**Keywords:** LED, Photodynamic therapy, Acne, Aging, Androgenetic alopecia, Alopecia

## Abstract

Benefit deriving from the use of light is known since ancient time, but, only in the last decades of twentieth century, we witnessed the rapid expansion of knowledge and techniques. Light-emitted diode (LED)-based devices represent the emerging and safest tool for the treatment of many conditions such as skin inflammatory conditions, aging, and disorders linked to hair growth. The present work reviews the current knowledge about LED-based therapeutic approaches in different skin and hair disorders. LED therapy represents the emerging and safest tool for the treatment of many conditions such as skin inflammatory conditions, aging, and disorders linked to hair growth. The use of LED in the treatment of such conditions has now entered common practice among dermatologists. Additional controlled studies are still needed to corroborate the efficacy of such kind of treatment.

## Introduction

Use of light as therapeutic approach is one of the oldest known methods to treat different health conditions, and its benefits are known since the ancient Egyptians, Chinese, and Indian populations [1–4]. Nevertheless, large use and well-known benefits for more than thousands of years, the scientific basis of phototherapy was laid at the beginning of twentieth century when the term “photodynamic therapy” (PDT) was coined by Oscar Raab and Herman von Tappeiner as referred to the chemical reaction in which oxygen is consumed following induction by a photosensitization process [5, 6]. This was followed in 1903 by the first reported use of artificial irradiation in phototherapy by a Danish physician, Niels Ryberg Finsen, winner of the Nobel Prize in Physiology or Medicine. In the same year, von Tappeiner and Jesionek reported the use of a combination of light and a topical substance, eosin, to treat skin tumors [1]. From its discovery till now, PDT is growing fast and significant progress has been made so far in light-based treatment of different disorders such as lung disease [7, 8], age-related degenerative processes of macula [9], urology [10–12], periodontal diseases [13], and different kinds of solid tumors [14]. Application of PDT in the dermatological field is characterized by the greatest use, and this is due not only to the easier accessibility of the skin for light exposure and photosensitizing topical applications but mainly to continuous advances in research.

Different kinds of PDT are currently available differing themselves as regards light source or photosensitizers used [15, 16]. Light sources directly influence the efficacy of treatment and include mainly low-level visible or near-infrared light from lasers (low-level laser therapy, LLLT) and light-emitted diodes (LEDs) [17]. Also, incandescent filament and gas discharge lamps are currently available [17].

LLLT has been the prevailing PDT for a long time [18] even if many limitations are reported for this approach: complicated clinical setting, wavelengths used, or area that can be covered by light. LEDs conveniently eliminate such limitations and, on the contrary, reveal as cheaper and more compact. Therefore, compared to lasers, LED power output is significantly lower resulting as less invasive and less potentially harmful to targeted tissues [19]. As many studies reported [20], LEDs result non-ablative and non-thermal and, especially when no photosensitizers are used, not damaging to skin and tissues. No common adverse side effects such as pain, swelling, peeling reported with other laser therapies have been reported from patients experiencing LED therapy.

Invented in 1962, LED was at the beginning unable to produce a significant biological activity. First beneficial effects for human health have been found by the National Aeronautics and Space Administration (NASA) with the development of LEDs producing a narrow spectrum of light in a non-coherent manner, able to deliver the appropriate wavelength and intensity required for the process. In the past 15 years, LED technology was continuously ameliorated. Red, blue, yellow, and near-infrared, also known as monochromatic infrared energy (MIRE), lights are today available.

LED therapy is nowadays a US Food and Drug Administration (FDA)-approved cosmetic procedure, in which observed effects include increased ATP production, modulation of intracellular oxidative stress, the induction of transcription factors, alteration of collagen synthesis, stimulation of angiogenesis, and increased blood flow [21]. LED biological effects are strongly influenced by light parameters as by clinical therapy [15].

The possibility to act on all of these parameters makes LED therapy highly flexible and adaptable for the treatment of different skin disorders; each one implicates different biological effects to be addressed.

Several studies reported effectiveness and safety of LED therapy in photo-aged skin [22–26]. Therefore, narrowband LED therapy using blue light reveals its efficacy and safety as additional therapy for mild to moderate acne [27]. LED therapy efficacy was also reported for instance in wound-healing [28, 29] as in psoriasis [30–32] and rosacea [33–36].

Recent studies [37] also demonstrated the antimicrobial effect of blue light. This antimicrobial effect is a non-thermal photochemical reaction involving the simultaneous presence of visible light, oxygen, and photosensitizer. Once photosensitization has been activated by the proper light source, chemical reactions are triggered leading to the production of various reactive oxygen species (ROS) [38]. Antimicrobial efficacy of PDT has been verified against a wide range of pathogens also in biofilm forms [39]. Therefore, use of blue light (405 nm) followed by treatment with red light (603 nm) is under investigation in the treatment of skin disorder involving microbial agents. Evidence on the efficacy of LED therapy for antimicrobial purposes also suggests its possible application in modulating skin microbiome.

This article will review the current LED-based therapeutic approach in different skin and hair disorders.

### Behind LED physiochemistry and photobiomodulation

A typical LED system is based on a semiconductor chip upon a reflective surface. When electricity runs through the system, light is produced. From a radiometric point of view, LED emission curve is in the form of a Lambertian pattern in which all the light is emitted at angles less than 90°.

The knowledge and definition of physical parameters are obligatory steps when setting-up PDT therapy. Maximization of LED therapy is strictly related to optimization of treatment parameters: (i) intensity and dosage, (ii) fluence rate, (iii) wavelength, (iv) pulsing or continuous mode, and (v) treatment duration [40]. Intensity or irradiance refers to the dose of energy delivered by the LED system per surface area of skin treated and is expressed in watts per square centimeter (W/cm^2^). The optimal clinical intensity or irradiance is considered to be around 50–100 mW/cm^2^.

**Fig. 1:**
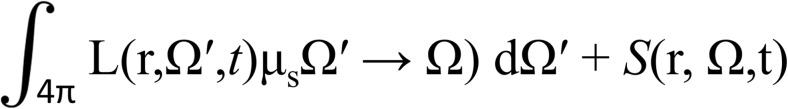
Radiation transport equation (RTE). *L (r, Ω)* is the radiant power transported at location *r* in a given direction *Ω* per unit solid angle per unit area perpendicular to that direction; *Ω* and *Ω′*are the propagation directions before and after elastic scattering; μ_s_ (Ω → Ω*′*) is the differential scattering coefficient; *S(r, Ω, t)* refers to the light source both internal and external

Another key part of the process is the definition of the optical properties of the tissues [15]. Once these have been defined, the fluence rate at any position for a given source specification can be calculated by mean of radiation transport equation (RTE) (Fig. 1) [41]. This equation describes light propagation to the site of treatment in a given direction per unit solid angle per unit area perpendicular to that direction. Since the resolution of this equation is not possible in almost all cases, three alternative approaches have been introduced [15]. Therefore, when setting up these kinds of physical evaluation, it is also important to consider the impact of the different geometries, such as the surface and the interstitial modality of irradiation, on the distribution of fluence rate [15].

Finding the appropriate combinations between the dose, the irradiance and the intensity of treatment are another important parameters to be considered to achieve optimal effects on the targeted tissues. Each skin conditions will require a specific evaluation of these parameters.

Different wavelengths can be produced depending on the composition of the semiconductor and LED system can deliver light either in continuous or in pulsed mode.

**Fig. 2:**
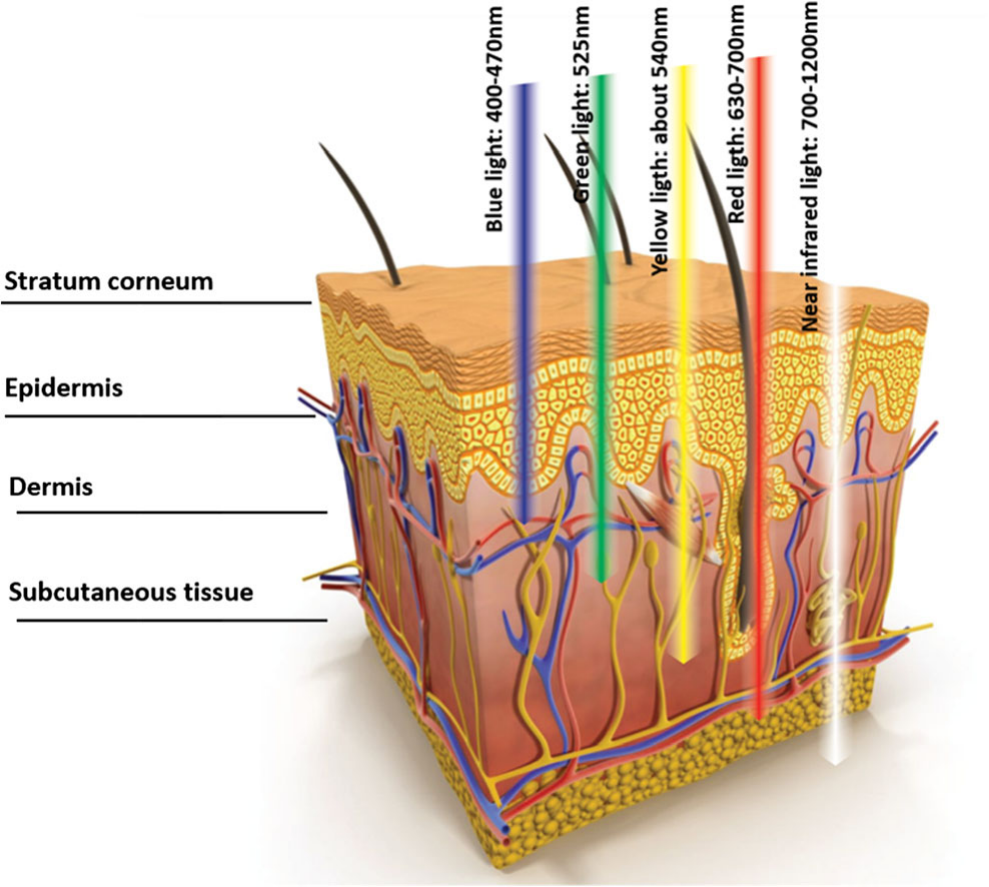
How different wavelengths penetrate the skin

Used wavelengths ranged from 400 to 1200 nm (Fig. 2); longer wavelengths are able to go deeper into tissues [42, 43]. Different cells and tissues absorb light at different wavelengths, and this is strictly related to the penetration that the wavelengths have to achieve.

Red light (630–700 nm) is able to reach dermis activating fibroblasts, increasing fibroblast growth factor expression as type 1 procollagen and matrix metalloproteinase-9 (MMP-9) [44].

Blue light (400–470 nm) has a lower potential for penetration and reveals useful for skin conditions in the epidermis layer of the skin [45]. Yellow light (about 540 nm) is effective in skin conditions involving redness, swelling, and other effects related to pigmentation [40]. Near-infrared light (700–1200 nm) reaches the maximum penetration in the skin; in vivo studies reveal its effectiveness in wound healing via angiogenesis stimulation [46].

Conflicting results are still reported as regards the best wave transmission system [47] even though there is some reported evidence showing a more favorable impact of pulsed mode on collagen de novo production by fibroblasts and a greater stimulatory effect on cell proliferation and oxidation [48, 49].

Establishing a time-dependent response and a precise working distance are others but not less mandatory parameters to ensure optimal results [40].

As a kind of PDT, LED acts on tissue and cells by photo-biomodulation. This process typically involves three key elements: a light source, a photosensitizing agent, and oxygen.

The light sources have to be chosen according to the capacity of the match both the activation spectrum of the photosensitizer and to generate the adequate power at the selected wavelength [17]. Once exposed to the chosen wavelengths, the photosensitizer comes to an excitation stage following two types of reactions, which led to free radical or singlet oxygen production (1O2). The last is highly active in biological systems and able to interfere with the mitochondrial electron transport chain in the cells via cytochrome c oxidase enzyme. Consequently, cells of the photostimulated tissue will increase the production of endogenous energy in the form of ATP and therefore will rapidly restore their integrity. By this process, LED therapy is able to stimulate fibroblasts, lymphocytes, keratinocytes, and melanocytes [49] and macrophage proliferation [50, 51].

Other observed effects include modulation of cell oxidation [52], anti-inflammatory effects [53], stimulation of angiogenesis and blood flow [54], the induction of transcription factors [55], antibacterial activity [42], and the alteration of collagen synthesis [43].

### Photosensitizers in LED therapy

Different types of photosensitizers are available for PDT. First-generation photosensitizers belong to the group of porphyrins. Porphyrin was approved by FDA in 1975 [55]. However, the use of this class of photosensitizers is limited to superficial tumor since they get excited only in the visible region. In order to overcome the above limitation, the second generation of photosensitizers has been developed as modified or substituted porphyrin. Among these, in 1999, FDA approved 5-aminolevulinic acid (ALA) and in 2004 its less polar methyl ester aminolevulinate (MAL) for dermatological indications [56, 57]. Both photosensitizers are prodrugs metabolized, inside the cell, to protoporphyrin IX [58–60]. This leads to endogenous porphyrin accumulation before light exposure. Besides their use in the PDT, these photosensitizers are also valuable markers for diagnosis of skin tumors [61]. In 1999, we reported the use of topical application of ALA for the identification of premalignant skin conditions, by means of fluorescence images. At the same times, this approach also reveals its utility in the clinical practice by reducing the number of biopsies required for the identification of malignant lesions [62].

Further photosensitizers are under study: (i) temoporfin, belonging to the chlorine family, with a higher light absorption at a longer wavelength (652 nm) in comparison with classic porphyrins [33] and (ii) indole-3-acetic acid (IAA) [63]. The third generation of photosensitizers is also under study. The development of this new class includes both the conjugation of photosensitizers with carrier bio-molecules and targeting peptides [16].

### LED therapy in inflammatory and auto-immune skin conditions

#### Acne vulgaris

Acne vulgaris is a multifactorial skin disorder associated with pilosebaceous unit inflammation [16, 64, 65]. Both oral and topical treatments are currently available even though they can be ineffective or poorly tolerated in some patients [66]. Some studies have suggested promising results for light therapies. During metabolic and reproductive processes, *Propionibacterium acnes* produces endogenous porphyrins, responsible for light absorption [67]. Evidence of acne improvement after sunlight exposure suggested the development of light-based therapy as a newer therapeutic approach.

Both red and blue lights reveal their efficacy for the treatment of acne vulgaris. In particular, some in vitro studies demonstrated a statistically significant inhibitory effect of red light (630 nm) on sebum production [68, 69].

Also, blue light (415 nm) showed a significant effect in acne treatment acting in a dose-dependent manner in reducing human sebocyte proliferation [67]. Many studies also reported the beneficial effects of blue light treatments in acne vulgaris via the alteration of skin microbiome [69–71]. PDT treatment can act directly on microorganism’s density (e.g., *P. acnes* density) [70] but also indirectly by modulating the immune response [71]. Our new research is evaluating the real effect of blue and red light (630 nm) on skin and scalp microbiome.

Other clinical trials also reported the efficacy of a combination of red and blue light in treating mild to moderate inflammatory acne lesions [72, 73].

In another study, Barolet and Boucher [74] reported inflammatory lesion reduction following a combined treatment for 4 weeks with LED (970 nm) and ALA-PDT + LED (630 nm) versus LED (630 nm) only. Combined treatment showed a 78% lesion reduction compared to 38% of LED only treatment. A more recent study by Zhang and collaborators [75] confirmed the efficacy of ALA-PDT and red light treatment in acne vulgaris.

#### Rosacea

Rosacea is an inflammatory skin condition characterized by flushing, facial erythema, dryness and burning of the skin, telangiectasia, vascular inflammation, inflammatory papules, and pustules and red or watery eyes [76]. Pathophysiology of Rosacea is strictly related to the abnormal expression of cathelicidins antimicrobial peptides, the elevated levels of stratum corneum tryptic enzymes (SCTE), and the expression of higher amounts of Toll-like receptor 2 (TLR2) in skin [73, 77]. Topical and oral therapies are currently available aimed at controlling Rosacea symptoms [78]. Unfortunately, these treatments do not carry a complete resolution and are not well tolerated in all patients. There is new evidence as regards the benefit of PDT and in particular LED therapies on Rosacea [33], even if more clinical trials are required. Recently, Bryld and Jemec showed the efficacy of MAL-PDT coupled with a red light on papulopustular lesions in Rosacea patients [34]. Another study by Lee and collaborator [35] reported the in vitro efficacy of LEDs at 630 and 940 nm on TLR2 and kallikreins (KLKs) in keratinocytes and rosacea-like mouse skin. Another in vitro study reported the efficacy of ALA-PDT against biofilm of *Staphylococcus aureus* [36].

#### Eczema

Atopic eczema or atopic dermatitis or simply eczema is a chronic, pruritic, inflammatory skin condition affecting up to 20% of children and 2–8% of adults [79], whom causes remain still unclear [80, 81]. Treatment of eczema by PDT represents a valid second-line therapy after non-pharmacological and topical measure failure.

Only one published randomized controlled trial has been found related to the use of LED therapy [82]. Patients treated with blue light (453 nm) for 4 weeks showed a 30% improvement of clinical manifestations of atopic dermatitis. Even if there is limited published evidence of LED efficacy for eczema, its anti-inflammatory effect is generally accepted and used in clinical practice as off-label treatment showing varying degrees of beneficial effect treatment for both kids and adults for moderate to severe conditions of eczema.

#### Psoriasis

Psoriasis is an immune-mediated inflammatory skin disorder affecting 2–3% of the population [83]. Since protoporphyrin IX (PpIX) is endogenously present in psoriatic conditions, it represents a potential target for photodynamic treatment [84]. Currently, three double-blind controlled studies reported the use of LED therapy in psoriatic subjects [85–87]. The first one reported a plaque erythema reduction of 33.9 and 26.7%, respectively, after comparing a 4-week treatment with blue light (420 nm) and red light (630 nm), respectively, at 60 J/cm^2^, 50 mW/cm^2^, 20 min. Compared to daily salicylic acid, LED therapy was less effective to reduce plaque desquamation, while more effective against erythema [85]. Since PpIX has a maximum absorption peak at 408 nm, it results more activated by blue light than red light and this reflects in the efficacy of the treatment.

The other two studies reported improvement of local psoriasis after blue light LED therapy (420 and 453 nm, respectively; 100 or 200 mW/cm^2^ of irradiance) in 4 weeks of treatment [86, 87]. Despite reported efficacy, more studies are currently needed for more precise recommendations about irradiance to be used.

### LED therapy as anti-aging and rejuvenation treatment

Skin aging is the result of intrinsic and environmental factors [88]. Aged and photo-damaged skin are characterized by a reduction in the synthesis of collagen and the simultaneous increase of matrix-metalloproteinase (MMP) expression. Skin rejuvenation treatments involve the use of retinoic acid, resurfacing by laser, chemical peels (trichloroacetic acid and CO_2_) [89, 90]. Other approaches include the use of injectable skin rejuvenation and dermal fillers [90] and polypeptides that have recently shown the ability to stimulate skin rejuvenation when topically applied [91]. More recently, autologous platelet-rich plasma (PRP) has attracted attention for skin rejuvenation [92–95] although the molecular mechanism of skin rejuvenation remains still largely unknown. Non-ablative skin rejuvenation by PDT is recently becoming a rather common therapeutic approach in skin rejuvenation thanks to its safety and effectiveness. Many in vitro and in vivo studies showed the ability of LED therapy to trigger skin collagen synthesis and to reduce MMP expression [49, 96, 97]. Rejuvenation effects have been reported followed by treatment with yellow LED (590 nm) on 900 patients [98]. Red light (660 nm) effects were also assessed in aged/photoaged individuals in a split-face single-blinded study by Barolet and collaborators [99]. This study showed that LED therapy is able to reverse collagen downregulation and MMP-1 upregulation, suggesting that the use of LED at 660 nm could represent a safe and effective collagen enhancement strategy. Evidence has been also reported indicating the higher efficacy of the combination of different wavelengths in LED therapy than monotherapy [40, 100]. Therefore, the use of blue light coupled with ALA-PDT showed improved elasticity, texture, pigmentation, and complexion of the skin [101–103]. In an in vivo study of 20 subjects [104], Zane and collaborators showed also a statistically significant improvement of skin rejuvenation following treatment with MAL-PDT and red light. The efficacy of this treatment has been also demonstrated in a larger study, involving 94 subjects [105]. LED therapy has also been successfully coupled with LLLT as adjuvant therapy for enhancing existing result from photo rejuvenation treatments [106].

### LED therapy in pre-cancerous and cancerous skin lesions

Pre-cancerous skin lesions refer to skin lesions with a certain degree of risk of progression to squamous cell carcinoma of the skin [107]. Actinic keratosis (AK) represents the most common pre-cancerous lesion encountered in clinical practice, developing after long-term exposure to the sun. Among differently available therapies for AK [108], photodynamic therapy is going to be an additional option. Therapy PDT has been recognized as effective in the treatment of AK at sites of poor healing or in case of poor response to other topical therapies by therapy guidelines [109, 110]. A randomized intra-individual study of face/scalp AK in 119 patients published by Morton and collaborators [111] compared LED therapy using MAL as photosensitizer to conventional cryotherapy. This study highlights a significantly higher rate of healing after PDT treatment and an equivalent response in non-responder-retreated subjects. Another study reported by Piacquadio and collaborators [112] reported 75% clearance of lesions in 77% of studied patients after treatment with a formulation containing 20% of ALA and blue light. Another randomized study showed the efficacy of narrowband red LED source coupled to BF-200 nano-emulsion [113]. Recent studies compared red light LED-PDT to daylight-PDT [114, 115]. Both studies demonstrated slightly higher clearance and recurrence rates with LED therapy.

PDT therapy is also considered a reasonable option for treatment, even though not as first-line, for small and superficial basal cell carcinoma (BCC). Use of red narrowband LED light has also been reported in the treatment of squamous cell carcinoma (SCC) in situ [116].

### LED therapy for hair loss disorders

**Fig. 3:**
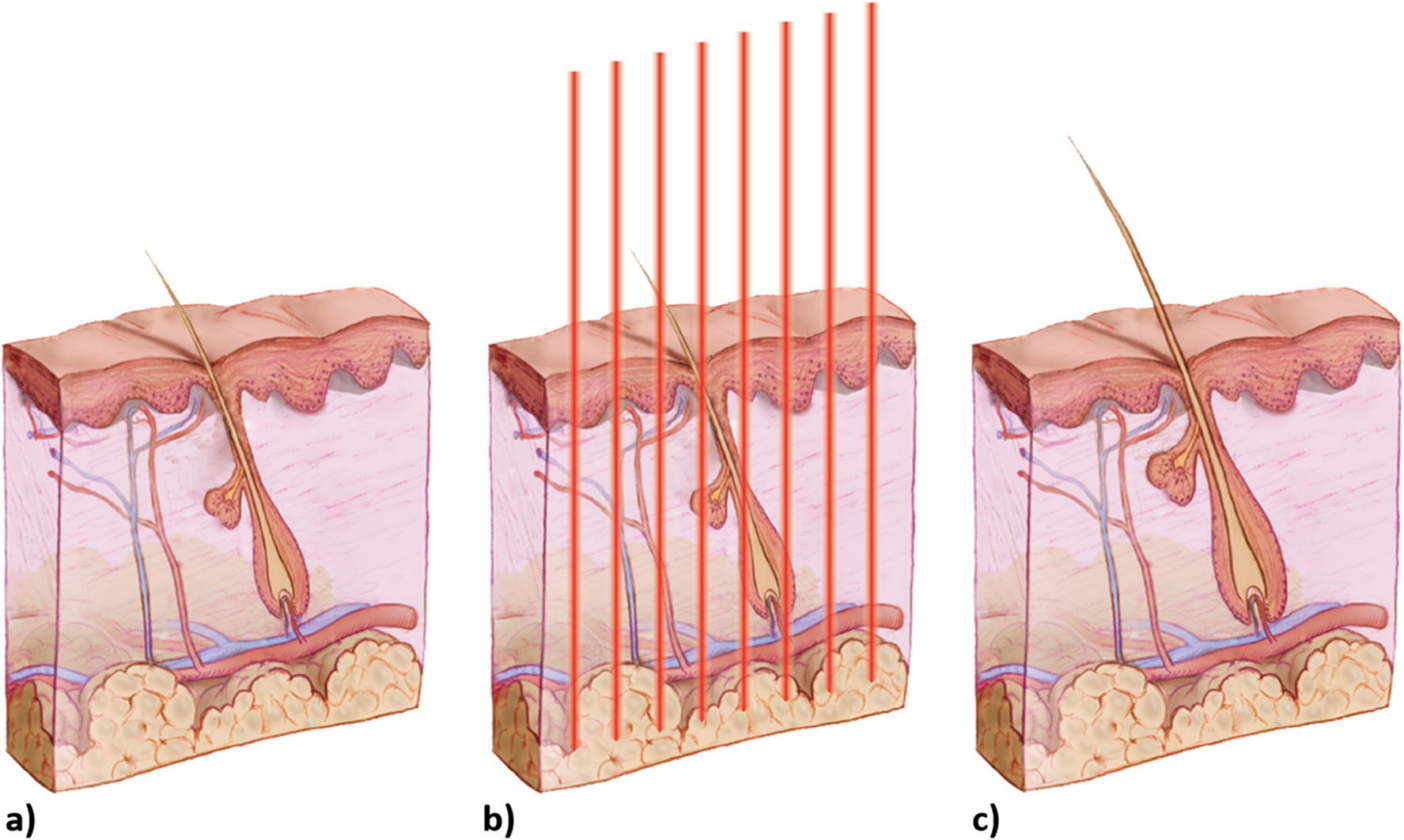
Representative image of LED therapy on androgenetic alopecia. **a** Miniaturized hair follicle; **b** treatment with red light (630 nm); healthy hair follicle

The efficacy of PDT for the treatment of hair loss is reported in several published studies [117]. Main reported evidence refers to LLLT as the most used light source [118–120]. In the 2007, FDA approved the first LLLT device (laser, 635 nm) for the treatment of hair loss, in particular for androgenetic alopecia. Following, in 2009, FDA approved similar device (laser, 655 nm) for alopecia, both in men and female. More recently also, LED therapy showed a real efficacy in the field of hair loss, especially therapies involving the use of red and infrared wavelengths [121, 122]. Today, both laser and LED devices have FDA approval for hair loss. In two studies reported from Lanzafame and collaborator [121, 122], 655 nm red light significantly improved hair counts both in men and women with androgenetic alopecia (Fig. 3). A more recent study [40] reported the effect of yellow LED device both on patients with androgenetic alopecia and alopecia areata. The efficacy of LED therapy by visible light has also been recognized as a valid adjuvant therapy in the recalcitrant form of alopecia areata [123].

Nowadays, no PDT studies are available for telogen effluvium, although the use of LLLT and especially LED has now entered common practice among dermatologists both in pre- and post-surgical periods. Also, the role of PDT and LED therapy in scarring alopecia should be further studied as a potential adjuvant treatment in the clinical management of cicatricial alopecia. ALA-PDT has been successfully used in the treatment of cutaneous Lichen Planus, as reported by many case reports [124]. PDT may act both on hyperproliferation of cells [125] and also by an immunomodulatory effect with increasing CD8+ reaction [126]. This evidence coupled with the first data on LP treatment encourages the use of LED therapy also in subjects with cicatricial alopecia such as Lichen Planopilaris.

## Limitations section

Despite its increasing efficacy and use in medical practice, knowledge on LED therapy remains still limited. Published studies often addressed a small number of patients (*n* < 20) and are difficult to compare each other since diversity in parameters used.

Therefore, as explained above, wavelength, irradiation, power density, and treatment time period can influence clinical outcomes at several degrees. Different devices, from different manufacturers, may present differences in light output and power densities. These limitations pose the need of future larger (patient sample *n* > 20) and more controlled studies in order to define LED therapy efficacy in different skin conditions, each of one presents specific parameters to be set up.

## Conclusion

PDT is an effective form of treatment for an increasing number of human conditions, ranging from cancer to several skin conditions. Benefit deriving from the use of light is known since ancient time, but only in the last decades of twentieth century, we witnessed the rapid expansion of knowledge and techniques. Recent improvements of the therapy are related especially to photosensitizer’s development and delivery systems. Nowadays, the use of LED-based devices represents the emerging and safest tool for the treatment of many conditions such as skin inflammatory conditions, aging, and disorders linked to hair growth. Although the use of LED in the treatment of hair disorders has now entered common practice, better controlled studies are still needed to corroborate its efficacy.

## Abbreviations

LED: Light-emitted-diodes
PDT: Photodynamic therapy
LLLT: Low-level laser therapy
FDA: US Food and Drug Administration
ALA: 5-aminolevulinic acid
MAL: Methyl ester aminolevulinate
ROS: Reactive oxygen species
MMP: Metalloproteinase
IAA: indole-3-acetic acid
PpIX: protoporphyrin IX
AK: Actinic keratosis
BCC: Basal cell carcinoma
SCC: Squamous cell carcinoma
LP: Lichen Planus
LPP: Lichen Planopilaris
FFA: Frontal fibrosing alopecia
FAPD: Fibrosing alopecia in a pattern distribution
VAS: Visual analog scale
AGA: Androgenic Alopecia

## References

[1] Ackroyd R, Kelty C, Brown N, Reed M (2001). The history of photodetection and photodynamic therapy. Photochem Photobiol.

[2] Daniell MD, Hill JS (1991). A history of photodynamic therapy. Aust NZ J Surg.

[3] Spikes JD, Berghausen RV, Jori G, Land EJ, Truscott TH (1985). Primary Photoprocesses in Biology and Medicine.

[4] Fitzpatrick TB, Pathak MA (1959). Historical aspects of methoxsalen and other furocoumarins. J Invest Dermatol.

[5] Raab O (1900). Uber die Wirkung fluoreszierender Stoffe auf Infusorien. Z Biol.

[6] Von Tappeiner H (1900). Uber die Wirkung fluoreszierender Stoffe auf Infusorien nach Versuchen von O. Raab. Muench Med Wochenschr.

[7] Ost D (2003). Photodynamic therapy in lung cancer. A review Methods Mol Med.

[8] Sutedja TG, Postmus PE (1996). Photodynamic therapy in lung cancer. A review. J Photochem Photobiol B Biol.

[9] Silva JN, Filipe P, Morliere P (2008). Photodynamic therapy: dermatology and ophthalmology as main fields of current applications in clinic. Biomed Mater Eng.

[10] Pinthus JH, Bogaards A, Weersink R, Wilson BC, Trachtenberg J (2006). Photodynamic therapy for urological malignancies: past to current approaches. J Urol.

[11] Juarranz A, Jaen P, Sanz-Rodriguez F, Cuevas J, Gonzalez S (2008). Photodynamic therapy of cancer. Basic principles and applications. Clin Transl Oncol.

[12] Jichlinski P (2006). Photodynamic applications in superficial bladder cancer: facts and hopes!. J Environ Pathol Toxicol Oncol.

[13] Chondros P, Nikolidakis D, Christodoulides N, Rossler R, Gutknecht N, Sculean A (2009). Photodynamic therapy as adjunct to non-surgical periodontal treatment in patients on periodontal maintenance: a randomized controlled clinical trial. Lasers Med Sci.

[14] Breskey JD, Lacey SE, Vesper BJ, Paradise WA, Radosevich JA, Colvard MD (2013). Photodynamic therapy: occupational hazards and preventative recommendations for clinical administration by healthcare providers. Photomed Laser Surg.

[15] Wilson BC, Patterson MS (2008 May 7) The physics, biophysics and technology of photodynamic therapy. Phys Med Biol 53:R61–R109 10.1088/0031-9155/53/9/R01 18401068

[16] Chilakamarthi U, Giribabu L (2017). Photodynamic therapy: past, present and future. Chem Rec.

[17] Brancaleon L, Moseley H (2002). Laser and non-laser light sources for photodynamic therapy. Lasers Med Sci.

[18] Hawkins D, Abrahamse H (2007). Phototherapy –a treatment modality for wound healing and pain relief. Afr J Biomed Res.

[19] Tong R, Kohane DS (2012). Shedding light on nanomedicine. WIREs Nanomed Nanobiotechnol.

[20] Ablon G (2018). Phototherapy with light emitting diodes: treating a broad range of medical and aesthetic conditions in dermatology. J Clin Aesthet Dermatol.

[21] Calderhead RG (2007). The photobiological basics behind light-emitting diode (LED) phototherapy. Laser Therapy.

[22] Lee SY, Chung EY, Park MY (2006). Blue and red light combination LED phototherapy for acne vulgaris in patients with skin phototype IV. Lasers Surg Med.

[23] Lowe N, Lowe P (2005). Pilot study to determine the efficacy of ALA-PDT photorejuvenation for thet reatment of facial ageing. J Cosmet Laser Ther.

[24] Kim JW (2005). Clinical trial of nonthermal 633 nm Omnilux LED array for renewal of photoaging: clinical surface profilometric results. Kor Soc Las Med Surg.

[25] Russell B, Reilly LR, Kellet N (2005). A study to determine the efficacy of combination LED light therapy (633 nm and 830 nm) in facial skin rejuvenation. J Cosmet Laser Ther.

[26] Babilas P, Kohl E, Maisch T (2006). In vitro and in vivo comparison of two different light sources for topical photodynamic therapy. Br J Dermatol.

[27] Ash C, Harrison A, Drew S, Whittall R (2015). A randomized controlled study for the treatment of acne vulgaris using high-intensity 414 nm solid state diode arrays. J Cosmet Laser Ther.

[28] Whelan HT, Smits RL, Buchman EV (2001). Effect of NASA light-emitting diode irradiation on wound healing. J Clin Laser Med Surg.

[29] Whelan HT, Buchmann EV, Dhokalia A (2003). Effect of NASA light-emitting diode irradiation on molecular changes for wound healing in diabetic mice. J Clin Laser Med Surg.

[30] Griffiths CE, van de Kerkhof P, Czarnecka-Operacz M (2017). Psoriasis and atopic dermatitis. Dermatol Ther (Heidelb).

[31] Ablon G (2010). Combination 830nm and 633nm light-emitting diode phototherapy shows promise in the treatment of recalcitrant psoriasis: preliminary findings. Photomed Laser Surg.

[32] Kleinpenning MM, Otero ME, van Erp PE (2012). Efficacy of blue light vs. red light in the treatment of psoriasis: a double-blind, randomized comparative study. J Eur Acad Dermatol Venereol.

[33] Triesscheijn M, Baas P, Schellens JH, Stewart FA (2006). Photodynamic therapy in oncology. Oncologist.

[34] Bryld LE, Jemec GB (2007). Photodynamic therapy in a series of rosacea patients. J Eur Acad Dermatol Venereol.

[35] Lee JB, Bae SH, Moon KR, Na EY, Yun SJ, Lee SC (2016). Light-emitting diodes downregulate cathelicidin, kallikrein and toll-like receptor 2 expressions in keratinocytes and rosacea-like mouse skin. Exp Dermatol.

[36] Li X, Guo H, Tian Q, Zheng G, Hu Y, Fu Y, Tan H (2013). Effects of 5-aminolevulinic acid-mediated photodynamic therapy on antibiotic-resistant staphylococcal biofilm: an in vitro study. J Surg Res.

[37] Dai T (2017). The antimicrobial effect of blue light: what are behind?. Virulence.

[38] Astuti SD, Wibowo R, Arif A, Triyana K (2017). Antimicrobial photodynamic effects of polychromatic light activated by magnetic fields to bacterial viability. J Int Dent Med Res.

[39] Ballester AR, Lafuente MT (2017). LED blue light-induced changes in phenolics and ethylene in citrus fruit: implication in elicited resistance against Penicillium digitatum infection. Food Chem.

[40] Opel DR, Hagstrom E, Pace AK (2015). Light-emitting diodes: a brief review and clinical experience. J Clin Aesthetic Dermatol.

[41] Wilson BC, Patterson MS (1986). The physics of photodynamic therapy. Phys Med Biol.

[42] Kalka K, Merk H, Mukhtar H (2000). Photodynamic therapy in dermatology. J Am Acad Dermatol.

[43] Simpson CR, Kohl M, Essenpreis M, Cope M (1998). Near-infrared optical properties of ex vivo human skin and subcutaneous tissues measured using the Monte Carlo inversion technique. Phys Med Biol.

[44] Jagdeo JR, Adams LE, Brody NI (2012). Transcranial red and near infrared light transmission in a cadaveric model. PLoS One.

[45] Friedmann DP, Goldman MP, Fabi SG, Guiha I (2014). The effect of multiple sequential light sources to activate Aminolevulinic acid in the treatment of actinic Keratoses: a retrospective study. J Clin Aesthetic Dermatol.

[46] Chaves ME de A, de Araújo AR, Piancastelli ACC, Pinotti M (2014). Effects of low-power light therapy on wound healing: LASER x LED. An Bras Dermatol.

[47] Al-Watban FA (2004). The comparison of effects between pulsed and CW lasers on wound healing. J Clin Laser Med Surg.

[48] Barolet D, Boucher A, Bjerring P (2005). In vivo human dermal collagen production following LED-based therapy: the importance of treatment parameters. Lasers Surg Med.

[49] Brondon P, Stadler I, Lanzafame RJ (2009). Pulsing influences photoradiation outcomes in cell culture. Lasers Surg Med.

[50] Bolton P, Young S, Dyson M (1990). Macrophage responsiveness to light therapy: a dose response study. Laser Ther.

[51] de Morais NC, Barbosa AM, Vale ML (2010). Anti-inflammatory effect of low-level laser and light-emitting diode in zymosan-induced arthritis. Photomed Laser Surg.

[52] Barolet DB (2008). Light-emitting diodes (LEDs) in dermatology. Semin Cutan Med Surg.

[53] Ghate VS, Ng KS, Zhou W (2013). Antibacterial effect of light emitting diodes of visible wavelengths on selected foodborne pathogens at different illumination temperatures. Int J Food Microbiol.

[54] Wunsch A, Matuschka K (2014). A controlled trial to determine the efficacy of red and near-infrared light treatment in patient satisfaction, reduction of fine lines, wrinkles, skin roughness, and intradermal collagen density increase. Photomed Laser Surg.

[55] Dougherty TJ, Grindey GB, Fiel R, Weishaupt KR, Boyle DG (1975). Photoradiation therapy. II. Cure of animal tumors with hematoporphyrin and light. J Natl Cancer Inst.

[56] Dolmans DE, Fukumura D, Jain RK (2003). Photodynamic therapy for cancer. Nat Rev Cancer.

[57] U.S. Food and Drug Administration. Center for Drug Evaluation and Research Metvixia NDA 21–415 approval letter. [(accessed on 28 February 2013)];2004 May 25; Retrieved 5 January 5 2013

[58] Angell-Petersen E, Sørensen R, Warloe T (2006). Porphyrin formation in actinic keratosis and basal cell carcinoma after topical application of methyl 5-aminolevulinate. J Invest Dermatol.

[59] Peng Q, Soler AM, Warloe T, Nesland JM, Giercksky KE (2001). Selective distribution of porphyrins in skin thick basal cell carcinoma after topical application of methyl 5-aminolevulinate. J Photochem Photobiol B.

[60] Fritsch C, Homey B, Stahl W, Lehmann P, Ruzicka T, Sies H (1998). Preferential relative porphyrin enrichment in solar keratoses upon topical application of delta-aminolevulinic acid methylester. Photochem Photobiol.

[61] Rosenthal I (1991). Phthalocyanines as photodynamic sensitizers. Photochem Photobiol.

[62] Cubeddu R, Pifferi A, Taroni P (1999). Time-gated and lifetime imaging techniques for the detection of skin tumors. Proc SPIE 3600, biomedical imaging: reporters, dyes, and instrumentation. 2.

[63] Wan MT, Lin JY (2014). Current evidence and applications of photodynamic therapy in dermatology. Clin Cosmet Investig Dermatol.

[64] Williams HC, Dellavalle RP, Garner S (2012). Acne vulgaris. Lancet.

[65] Tripathi SV, Gustafson CJ, Huang KE, Feldman SR (2013). Side effects of common acne treatments. Expert Opin Drug Saf.

[66] Bhardwaj S, Rohrer TE, Arndt K (2005). Lasers and light therapy for acne vulgaris. Semin Cutan Med Surg.

[67] Jung YR, Kim SJ, Sohn KC (2015). Regulation of lipid production by light-emitting diodes in human sebocytes. Arch Dermatol Res.

[68] Smith KR, Thiboutot DM (2008). Sebaceous gland lipids: friend or foe?. J Lipid Res.

[69] Charakida A, Seaton ED, Charakida M, Mouser P, Avgerinos A, Chu AC (2004). Phototherapy in the treatment of acne vulgaris: what is its role?. Am J Clin Dermatol.

[70] Noborio R, Nishida E, Kurokawa M (2007). Morita a. A new targeted blue light phototherapy for the treatment of acne. Photodermatol Photoimmunol Photomed.

[71] Thiboutot D, Gollnick H, Bettoli V et al (2009) Global alliance to improve outcomes in acne. New insights into the management of acne: an update from the global alliance to improve outcomes in acne group. J Am Acad Dermatol 60(Suppl):S1–S50 10.1016/j.jaad.2009.01.019 19376456

[72] Lee SY, You CE, Park MY (2007). Blue and red light combination LED phototherapy for acne vulgaris in patients with skin Phototype IV. Lasers Surg Med.

[73] Kwon HH, Lee JB, Yoon JY (2013). The clinical and histological effect of home-use, combination blue-red LED phototherapy for mild-to-moderate acne vulgaris in Korean patients: a double-blind, randomized controlled trial. Br J Dermatol.

[74] Barolet D, Boucher A (2010). Radiant near infrared light emitting diode exposure as skin preparation to enhance photodynamic therapy inflammatory type acne treatment outcome. Lasers Surg Med.

[75] Zhang L, Wu Y, Zhang Y (2017). Topical 5-aminolevulinic photodynamic therapy with red light vs intense pulsed light for the treatment of acne vulgaris: a spilit face, randomized, prospective study. Dermatoendocrinol.

[76] Maier LE (2011). Rosacea: advances in understanding pathogenesis and treatment. J Clin Invest.

[77] Cribier B (2011). Pathophysiology of rosacea: redness, telangiectasia, and rosacea. Ann Dermatol Venereol.

[78] Feldman SR, Huang WW, Huynh TT (2014). Current drug therapies for rosacea: a chronic vascular and inflammatory skin disease. J Manag Care Spec Pharm.

[79] Wollenberg A, Barbarot S, Bieber T, Christen-Zaech S, Deleuran M, Fink-Wagner A, Gieler U, Girolomoni G, Lau S, Muraro A, Czarnecka-Operacz M, Schäfer T, Schmid-Grendelmeier P, Simon D, Szalai Z, Szepietowski JC, Taïeb A, Torrelo A, Werfel T, Ring J (2018). European dermatology forum (EDF), the European academy of dermatology and venereology (EADV), the European academy of allergy and clinical immunology (EAACI), the European task force on atopic dermatitis (ETFAD), European Federation of Allergy and Airways Diseases Patients’ associations (EFA), the European Society for Dermatology and Psychiatry (ESDaP), the European Society of Pediatric Dermatology (ESPD), global allergy and asthma European network (GA2LEN) and the European Union of medical specialists (UEMS). Consensus-based European guidelines for treatment of atopic eczema (atopic dermatitis) in adults and children: part II. J Eur Acad Dermatol Venereol.

[80] Friedmann PS (2002). The pathogenesis of atopic eczema. Hosp Med.

[81] Pyun BY (2015). Natural history and risk factors of atopic dermatitis in children. Allergy Asthma Immunol Res.

[82] Keemss K, Pfaff SC, Born M, Liebman NJ, Merk HF, von Felbert V (2016). Prospectiv e, randomized study on the efficacy and safety of local UV-freeblue light treatment of eczema. Dermatology.

[83] Zhang P, Wu MX (2018). A clinical review of phototherapy for psoriasis. Lasers Med Sci.

[84] Bissonnette R, Zeng H, McLean DI (1998). Psoriatic plaques exhibit red autofluorescence that is due to protoporphyrin IX. J Invest Dermatol.

[85] Kleinpenning M, Otero M, van Erp P, Gerritsen M, van de Kerkhof P (2012). Ef®cacy of blue light vs. red light in the treatment of psoriasis: a double-blind, randomized comparative study. J Eur Acad Dermatol Venereol.

[86] Pfaff S, Liebmann J, Born M, Merk HF, Von Felbert V (2015). Prospective randomized long-term study on the ef®cacy and safety of UV-free blue light for treating mild psoriasis vulgaris. Dermatology (Basel, Switzerland).

[87] Weinstabl A, Hoff-Lesch S, Merk HF, von Felbert V (2011). Prospective randomized study on the efficacy of blue light in the treatment of psoriasis vulgaris. Dermatology (Basel, Switzerland).

[88] Farage MA, Miller KW, Elsner P, Maibach HI (2008). Intrinsic and extrinsic factors in skin ageing: a review. Int J Cosmet Sci.

[89] Baumann L (2007). Skin ageing and its treatment. J Pathol.

[90] Ganceviciene R, Liakou AI, Theodoridis A, Makrantonaki E, Zouboulis CC. Skin anti-aging ] strategies. Dermato-endocrinology 2012;4:308–319 10.4161/derm.22804 PMC3583892 23467476

[91] Lupo MP, Cole AL (2007). Cosmeceutical peptides. Dermatol Ther.

[92] Kim DH, Je YJ (2011). Kim CDet al. Can platelet-rich plasma be used for skin rejuvenation? Evaluation of effects of platelet-rich plasma on human dermal fibroblast. Ann Dermatol.

[93] Krasna M, Domanovic D, Tomsic A, Svajger U, Jeras M (2007). Platelet gel stimulates proliferation of human dermal fibroblasts in vitro. Acta Dermatovenerol Alp Panonica Adriat.

[94] Lucarelli E, Beccheroni A, Donati D, Sangiorgi L, Cenacchi A, Del Vento AM (2003). Platelet-derived growth factors enhance proliferation of human stromal stem cells. Biomaterials.

[95] Kanno T, Takahashi T, Tsujisawa T, Ariyoshi W, Nishihara T (2005). Platelet-rich plasma enhances human osteoblast-like cell proliferation and differentiation. J Oral Maxillofac Surg.

[96] Weiss RA, McDaniel DH, Geronemus R (2005). Clinical trial of a novel non-thermal LED array for reversal of photoaging: clinical, histologic, and surface profilometric results. Lasers Surg Med.

[97] Lee SY, Park KH, Choi JW (2007). A prospective, randomized, placebocontrolled, double-blinded, and split-face clinical study on LED phototherapy for skin rejuvenation: clinical, profilometric, histologic, ultrastructural, and biochemical evaluations and comparison of three different treatment settings. J Photochem Photobiol B.

[98] Weiss RA, McDaniel DH, Geronemus RG (2005). Clinical experience with light-emitting diode (LED) photomodulation. Dermatol Surg.

[99] Barolet D, Roberge CJ, Auger FA, Boucher A, Germain L (2009). Regulation of skin collagen metabolism in vitro using a pulsed 660 nm LED light source: clinical correlation with a single-blinded study. J Invest Dermatol.

[100] Jagdeo J, Austin E, Mamalis A, Wong C, Ho D, Siegel DM (2018) Light-emitting diodes in dermatology: a systematic review of randomized controlled trials. Lasers Surg Med. 10.1002/lsm.22791 10.1002/lsm.22791 PMC6099480 29356026

[101] Gold MH (2002). The evolving role of aminolevulinic acid hydrochloride with photodynamic therapy in photoaging. Cutis.

[102] Clementoni MT, B-Roscher M, Munavalli GS (2010). Photodynamic photorejuvenation of the face with a combination of microneedling, red light, and broadband pulsed light. Lasers Surg Med.

[103] Touma D, Yaar M, Whitehead S, Konnikov N, Gilchrest BA (2004). A trial of short incubation, broad-area photodynamic therapy for facial actinic keratoses and diffuse photodamage. Arch Dermatol.

[104] Zane C, Capezzera R, Sala R, Venturini M, Calzavara-Pinton P (2007). Clinical and echographic analysis of photodynamic therapy using methylaminolevulinate as sensitizer in the treatment of photodamaged facial skin. Lasers Surg Med.

[105] Palm MD, Goldman MP (2011). Safety and efficacy comparison of blue versus red light sources for photodynamic therapy using methyl aminolevulinate in photodamaged skin. J Drugs Dermatol.

[106] Calderhead RG, Vasily DB (2016). Low level light therapy with light-emitting diodes for the aging face. Clin Plast Surg.

[107] Dodds A, Chia A, Shumack S (2014). Actinic keratosis: rationale and management. Dermatol Ther.

[108] Dréno B, Amici JM, Basset-Seguin N, Cribier B, Claudel JP, Richard MA (2014). AKTeam™. Management of actinic keratosis: a practical report and treatment algorithm from AKTeam™ expert clinicians. J Eur Acad Dermatol Venereol.

[109] Stockfleth E et al. Guidelines on actinic keratosis. European Dermatology Forum: http://www.euroderm.org/edf/images/stories/guidelines/guideline_Management_Actinic_Keratoses-update2011.pdf

[110] De Berker D, McGregor J, Hughes B (2007). Guidelines for the management of actinic keratosis. Br J Dermatol.

[111] Morton C, Campbell S, Gupta G (2006). Intraindividual, right-left comparison of topical methyl aminolaevulinate-photodynamic therapy and cryotherapy in subjects with actinic keratoses: a multicentre, randomized controlled study. Br J Dermatol.

[112] Piacquadio DJ, Chen DM, Farber HF (2004). Photodynamic therapy with aminolevulinic acid topical solution and visible blue light in the treatment of multiple actinic keratoses of the face and scalp: investigator-blinded phase 3 multicenter trials. Arch Dermatol.

[113] Szeimies RM, Radny P, Sebastian M (2010). Photodynamic therapy with BF-200 ALA for the treatment of actinic keratosis: results of a prospective, randomized, double-blind, placebo-controlled phase III study. Br J Dermatol.

[114] Čarija A, Puizina-Ivić N, Vuković D, Mirić Kovačević L, Čapkun V (2016). Single treatment of low-risk basal cell carcinomas with pulsed dye laser-mediated photodynamic therapy (PDL-PDT) compared with photodynamic therapy (PDT): a controlled, investigator-blinded, intra-individual prospective study. Photodiagn Photodyn Ther.

[115] Fernandez-Guarino M, Harto A, Jaen P (2012) Pulsed dye laser does not seem as effective as red light in basal cell carcinoma mal-pdt: a small pilot study. J Skin Cancer 2012:396481 10.1155/2012/396481 PMC3503397 23209908

[116] Calzavara-Pinton PG, Venturini M, Sala R (2008). Methylaminolaevulinate-based photodynamic therapy of Bowen's disease and squamous cell carcinoma. Br J Dermatol.

[117] Dodd EM, Winter MA, Hordinsky MK, Sadick NS, Farah RS (2017). Photobiomodulation therapy for androgenetic alopecia: a clinician's guide to home-use devices cleared by the federal drug administration. J Cosmet Laser Ther.

[118] Avram MR, Leonard RT, Epstein ES, Williams JL, Bauman AJ (2007). The current role of laser/light sources in the treatment of male and female pattern hair loss. J Cosmet Laser Ther.

[119] Leavitt M, Charles G, Heyman E, Michaels D (2009). HairMax LaserComb laser phototherapy device in the treatment of male androgenetic alopecia: a randomized, double-blind, sham device-controlled, multicentre trial. Clin Drug Investig.

[120] Avram MR, Rogers NE (2010). The use of low-level light for hair growth: part I. J Cosmet Laser Ther.

[121] Lanzafame RJ, Blanche RR, Bodian AB, Chiacchierini RP, Fernandez-Obregon A, Kazmirek ER (2013). The growth of human scalp hair mediated by visible red light laser and LED sources in males. Lasers Surg Med.

[122] Lanzafame RJ, Blanche RR, Chiacchierini RP, Kazmirek ER, Sklar JA (2014). The growth of human scalp hair in females using visible red light laser and LED sources. Lasers Surg Med.

[123] Tzung TY, Chen CY, Tzung TY, Kao FJ, Chen WC (2009). Infrared irradiation as an adjuvant therapy in recalcitrant alopecia areata. Dermatol Surg.

[124] Atzmony L, Reiter O, Hodak E, Gdalevich M, Mimouni D (2016). Treatments for cutaneous lichen planus: a systematic review and meta-analysis. Am J Clin Dermatol.

[125] Amo T, Kawanishi N, Uchida M (2009). Mechanism of cell death by 5-aminolevulinic acid-based photodynamic action and its enhancement by ferrochelatase inhibitors in human histiocytic lymphoma cell line U937. Cell Biochem Funct.

[126] Edström DW, Porwit A, Ros AM (2001). Photodynamic therapy with topical 5-aminolevulinic acid for mycosis fungoides: clinical and histological response. Acta Derm Venereol.

